# Supplementary material for: Silica-Coated Magnetic Iron Oxide Nanoparticles Grafted onto Graphene Oxide for Protein Isolation
Source: Nanomaterials (Basel). 2020 Jan 8;10(1):117. doi: 10.3390/nano10010117 (PMC7022723; doi:10.3390/nano10010117)
Supplement: Supplementary file 1 [file nanomaterials-10-00117-s001.pdf]

Silica coated magnetic nanoparticles grafted graphene oxide for protein separation

Xuan-Hung Pham[1], Eunil Hahm[1], Hyung-Mo Kim [1], Byung Sung Son[1], Tuong An Tran Thi[2], Dinh Quan Nguyen[2], and Bong-Hyun Jun[1,*]

1 Department of Bioscience and Biotechnology, Konkuk University, Seoul 05029, Republic of Korea;

2 Laboratory of biofuel and biomass research, VNU-HCMU University of Technology, Ho Chi Minh, Viet Nam.

phamricky@gmail.com (X-H.P.); greenice@konkuk.ac.kr (E.H); hmkim0109@konkuk.ac.kr (H-M.K.); imsonbs@konkuk.ac.kr (B.S.S.); trantuongan81@gmail.com (T.A.T.T.); ndquan@hcmut.edu.vn (D.Q.N); bjun@konkuk.ac.kr (B.H.J.)

* Correspondence: bjun@konkuk.ac.kr; Tel.: +82-2-450-0521 (B.H.J.)

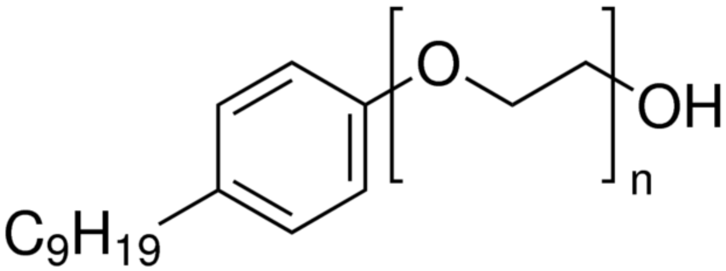

Figure S1. Molecular structure of Igepal®CO-520.

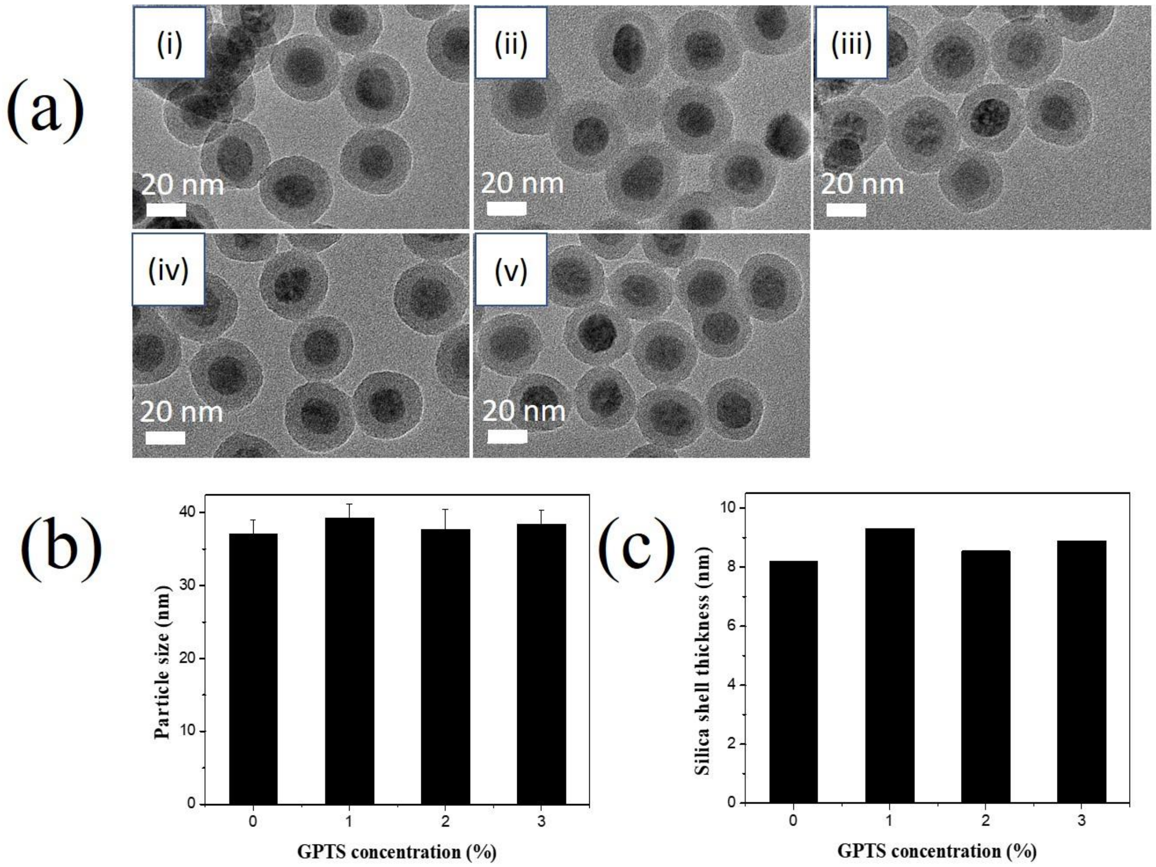

Figure S2. Effects of GPTS concentration on the silica coating of the magnetic nanoparticles. (a) TEM images (b) particle size and (c) silica shell thickness of MNP@SiO_2 synthesized at different GPTS concentration: (i) 0 %, (ii) 1%, (iii) 2%, (iv) 3%, and (v) 5% in the presence of 0.3 mL Igepal®CO-520, 5 mg MNP, 100 μL TEOS and 100 μL NH_4OH.

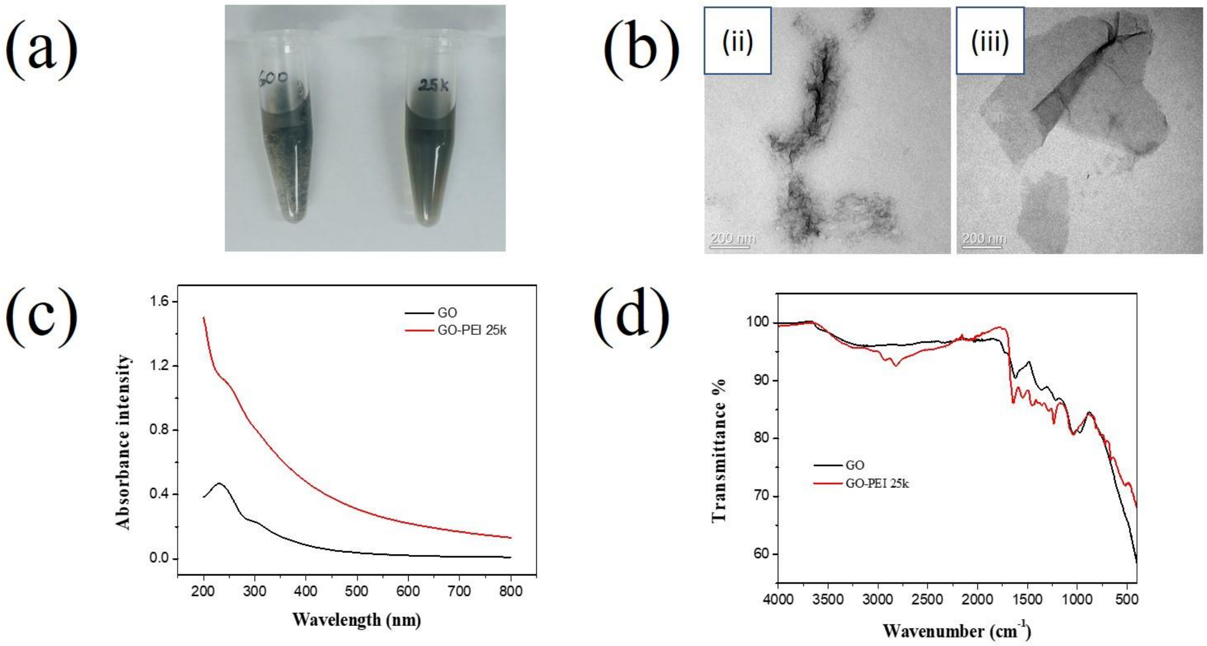

Figure S3. (a) Optical images of GO incubated with PEI 600, PEI 25k. (b) TEM images, (c) UV-Vis absorbance and (d) ATR-FTIR of (i) GO, (ii) PEI 25k grafted GO.

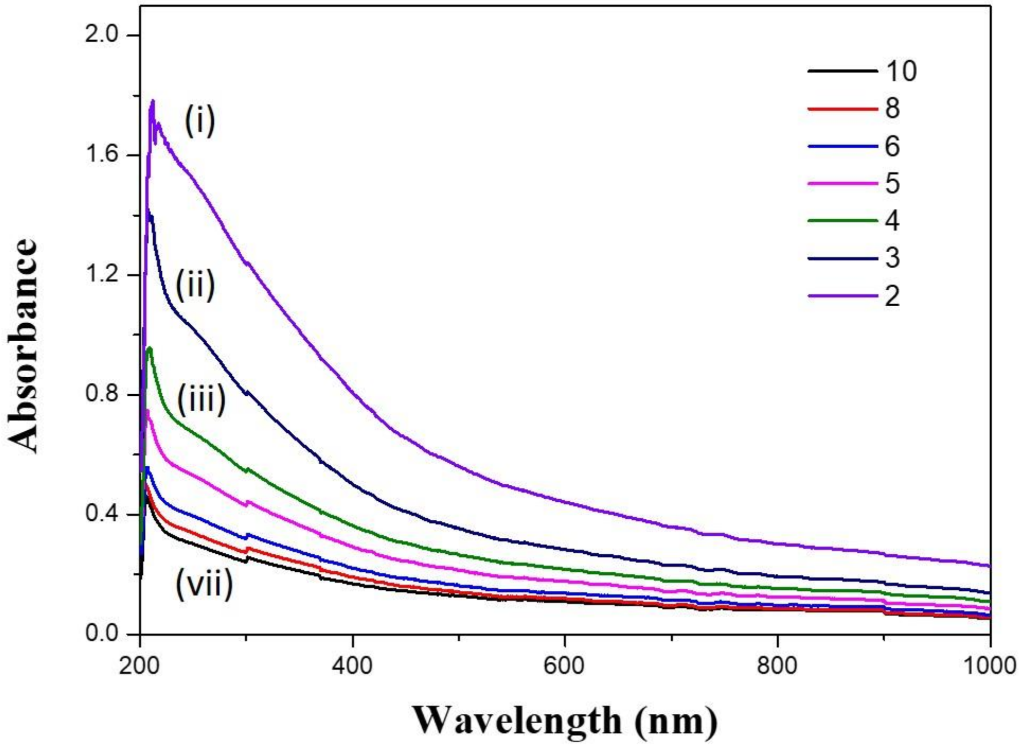

Figure S4. Absorbance spectra of GO/MNP@SiO_2 with various ratio of PEI grafted GO and GPTS coated MNP@SiO_2-GPTS. (i) 2:1, (ii) 3:1, (iii) 4:1, (iv) 5:1%, (v) 6:1, (vi) 8:1 and (vii) 10:1.

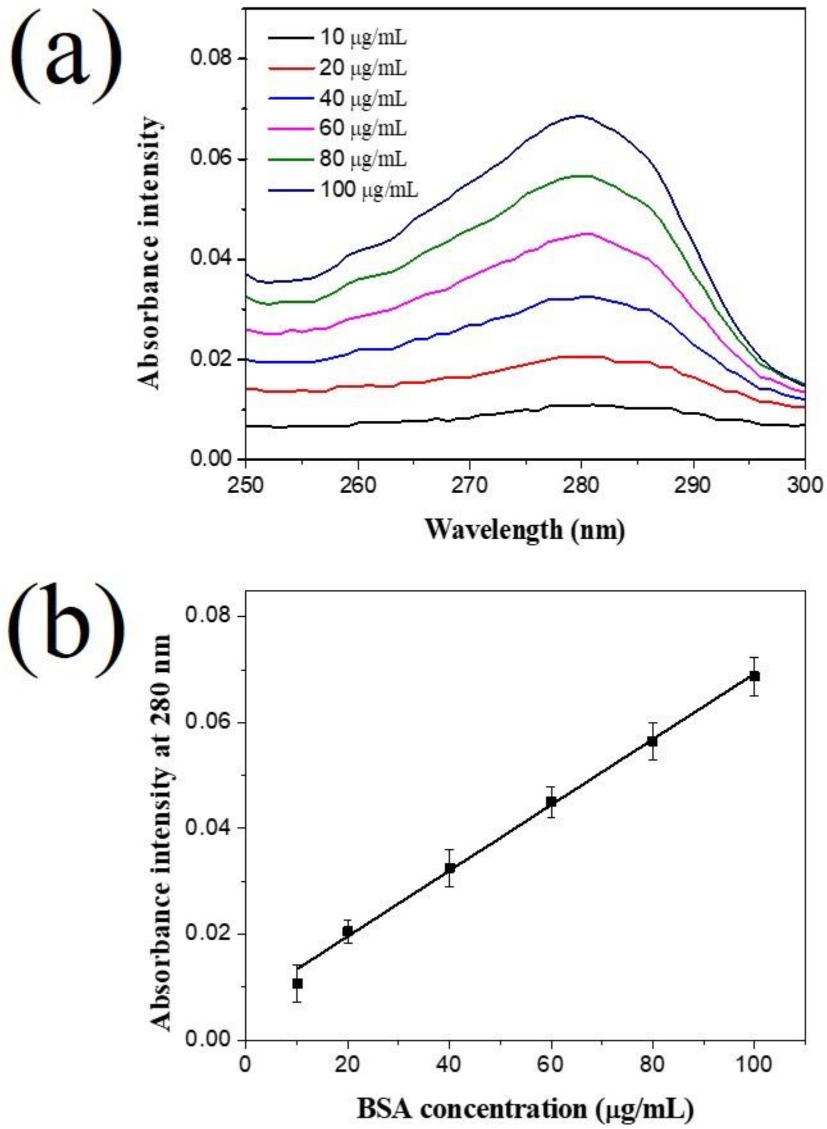

Figure S5. (a) Absorbance spectra and (b) calibration curves of BSA protein at different concentration.
